# Cryopreservation of *In Vitro*-Produced Early-Stage Porcine Embryos in a Closed System

**DOI:** 10.1089/biores.2015.0012

**Published:** 2015-05-01

**Authors:** Hongsheng Men, Lee D. Spate, Clifton N. Murphy, Randall S. Prather

**Affiliations:** ^1^ Department of Veterinary Pathobiology, University of Missouri, Columbia, Missouri.; ^2^ Division of Animal Sciences, Animal Sciences Research Center, University of Missouri, Columbia, Missouri.; ^3^ National Swine Resource and Research Center, University of Missouri, Columbia, Missouri.

**Keywords:** closed system, cryopreservation, porcine early-stage embryos, swine models

## Abstract

Cryostorage of porcine embryos in a closed pathogen-free system is essential for the maintenance and safeguard of swine models. Previously, we reported a protocol for the successful cryopreservation of porcine embryos at the blastocyst stage in 0.25 mL ministraws. In this experiment, we aimed at developing a protocol to apply the same concept for the cryopreservation of early-stage porcine embryos. Porcine embryos from day 2 through day 4 were delipidated by using a modified two-step centrifugation method and were then cryopreserved in sealed 0.25 mL straws by using a slow cooling method. Control groups included open pulled straw (OPS) vitrified embryos after delipidation and noncryopreserved embryos without delipidation. There were no significant differences in cryosurvival between embryos frozen in 0.25 mL straws and OPS vitrified embryos across all the stages (two cell to morula) examined (*p*>0.05). Similarly, in all groups examined, the blastocyst rates were not different between the two cryopreserved groups. However, the blastocyst rates from the cryopreserved groups were significantly lower than the noncryopreserved controls (*p*<0.05). This experiment demonstrated that early-stage porcine embryos can survive cryopreservation in a closed system by using a slow cooling method at a comparable rate to those vitrified by using an ultrarapid cooling method (*p*>0.05). However, the developmental competence was significantly reduced after cryopreservation compared to noncryopreserved embryos. Further research is needed to optimize the protocol to improve the developmental potential of cryopreserved early-stage porcine embryos in sealed straws.

## Introduction

Pigs have increasingly gained popularity for biomedical research as models of human diseases and health and as potential tissue and organ donors for xenotransplantation due to their similarities in structure and function to humans.^1–3^ Meanwhile, the newly developed genomic editing tools, such as the clustered regularly interspaced short palindromic repeats (CRISPR) and CRISPR-associated protein 9 (CRISPR/Cas9) system, make genetic modifications in pigs more efficient and cost-effective.^4,5^ Consequently, the number of swine models is expected to increase dramatically over the next few years. Therefore, there is an unprecedented need for user-friendly protocols that are efficient for the cryopreservation of sperm and embryos to safeguard these valuable models. Sperm and embryos from animal models are required to be in a closed system to keep germplasm from potential contamination with pathogens from various sources during cryostorage and transportation.^6–8^ However, due to the exceeding sensitivity of porcine embryos to low temperature, the open vitrification systems, such as Cryotop, metal mesh, minimal volume cooling, open pulled straws (OPSs), and hollow fibers, which can provide ultrafast cooling rates, have been employed for their efficient cryopreservation.^9–13^ Currently, only blastocysts efficiently survive cryopreservation by vitrification in sealed straws and maintain an intact zona pellucida.^14^ Development of protocols for the cryopreservation of early-stage porcine embryos in a closed system will provide flexibility for the maintenance of swine models; for example, when animal models have a serious health issue that necessitates collection at an early stage of development. It will also offer flexibility for mutant model creation by providing a method to safely store surplus genetically manipulated embryos or when there are no synchronized surrogates available.

However, the developmental competence of early-stage porcine embryos is significantly compromised when they are vitrified in a closed system by using the protocol described earlier.^14^ The compromised developmental potential is probably due to the stage-dependent sensitivity of porcine embryos to low temperature, as earlier stage embryos are more sensitive to low temperature than later stage embryos.^15^ The longer exposure of embryos to a vitrification solution due to the slower cooling rate in 0.25 mL straws increases the toxic effects of the high concentrations of cryoprotectants on embryos.^16,17^ There is only one report of live birth resulting from cryopreservation of early-stage porcine embryos in a closed system by using the slow cooling method after mechanical dilapidation.^18^

In addition to a closed system, embryos from animal models also need to maintain an intact zona pellucida during cryopreservation to reduce the risk of pathogen transmission.^19^ Previously, a noninvasive approach was used to disassociate lipid droplets from blastomeres in porcine four- to eight-cell-stage embryos through slight modifications of a centrifugation-based delipidation approach.^18,20^ This approach takes advantage of the large perivitelline spaces in four- to eight-cell-stage embryos to permit a complete disassociation of lipid droplets from blastomeres. However, porcine embryos at certain stages (i.e., zygotes and morula) have small perivitelline spaces and it is difficult to achieve complete delipidation by using this approach. Therefore, in this experiment, porcine early embryos were subjected to a two-step centrifugation treatment: the first-step is to polarize intracellular lipids at physiological osmolarity, whereas the second step is to completely disassociate lipid droplets from blastomeres by using hypertonic treatment to temporarily create larger perivitelline spaces by shrinking the blastomeres while applying centrifugal force.^21,22^

As mentioned above, vitrification of early-stage porcine embryos in 0.25 mL straws compromised their subsequent developmental potential. Therefore, in this experiment, embryos were cryopreserved by using a slow cooling method. Two hypotheses were tested in this experiment: (1) intracellular lipids from early-stage porcine embryos can be effectively externalized by a two-step centrifugation treatment and (2) the cryosurvival and *in vitro* developmental competence of early-stage porcine embryos cryopreserved by using slow cooling is comparable to vitrification at an ultrarapid cooling rate. We used embryos generated from *in vitro-*matured and *in vitro-*fertilized oocytes from a local slaughterhouse to test these hypotheses. Embryos without delipidation and cryopreservation served as noncryopreserved controls.

## Materials and Methods

### Ethics statement

Boars used for semen collection were housed in the Division of Animal Science facilities at the University of Missouri. The protocol was approved by the Animal Care and Use Committee of the University of Missouri. Ovaries were collected at a United States Department of Agriculture-inspected abattoir.

### Oocyte maturation, fertilization, and embryo culture

All the chemicals were obtained from Sigma Aldrich Co (St Louis, MO) unless otherwise stated. Immature porcine oocytes were purchased from ART, Inc. (Madison, WI). After 22 h of maturation in a Phase I medium, oocytes were transferred into a Phase II medium in Nunc four-well culture plates for an additional 20–22 h of maturation. At the end of maturation, cumulus cells were stripped off from matured oocytes by vortexing. Cumulus-free matured oocytes, in groups of 30, were then fertilized in 100 μL of modified Tris-buffered medium (mTBM) drops by using frozen–thawed sperm at a concentration of 5×10^5^ sperm/mL, as previously described.^23^ After 6 h of sperm–oocyte coculture, groups of 30 presumptive zygotes were cultured in four-well Nunc plates with each well containing 500 μL PZM-3 supplemented with 3 mg/mL fatty acid-free bovine serum albumin for subsequent development.^24^ Maturation, fertilization, and embryo culture were conducted in an incubator at 38.5°C with 5% CO_2_ and maximal humidity in air.

### Externalization of intracellular lipids

In this experiment, day 2 (day 0=fertilization) to day 4 porcine embryos (four-cell to morula) were subjected to a two-step centrifugation with porcine embryos in solutions of two different osmolalities to achieve a complete externalization of lipid droplets from blastomeres. The first step was to polarize the lipid droplets to one side of the embryos at physiological osmolality by centrifugation and the second step was to completely separate the polarized lipid droplets from blastomeres by using centrifugation in a hypertonic solution to create a temporary large perivitelline space, as detailed in the Experiment Design section.

### Embryo cryopreservation and thawing/warming

Porcine embryos at various stages were cryopreserved by either slow cooling or OPS vitrification. For slow cooling, embryos were cryopreserved in 0.25 mL straws, as previously described, with modifications.^25^ Briefly, delipidated embryos were equilibrated in 1.5 M 1,2-propanediol (PROH) in TCM199-HEPES+10% fetal bovine serum (FBS) for 15 min. After equilibration, embryos were transferred into 1.5 M PROH in TCM199-HEPES supplemented with 0.1 M sucrose and then loaded into 0.25 mL straws. Straws were directly placed into a programmable freezer (Freeze Control; Cryologic, Victoria, Australia), which was precooled to −6.5°C. Straws were seeded by touching the top portion of the straws with a forceps precooled in liquid nitrogen (LN_2_). Ten minutes after seeding, embryos were cooled at a rate of 0.3°C/min to −30°C and then the straws were directly plunged into LN_2_. After at least 1 day in storage in LN_2_, straws were thawed in a 30°C water bath. Rehydration of the embryos was conducted by exposing embryos sequentially to 0.75 M PROH+0.2 M sucrose in TCM199-HEPES+10% FBS for 10 min, 0.2 M sucrose in TCM199-HEPES+10% FBS for 5 min, and then in TCM199-HEPES+10% FBS for 10 min. Thawed embryos were examined under a dissecting microscope (Olympus America, Center Valley, PA). Embryos with intact plasma membrane and zona pellucida were classified as viable embryos and were cultured in groups of 30 in 500 μL PZM-3 supplemented with 10% FBS in Nunc four-well plates covered with mineral oil in an incubator maintained at 38.5°C, 5% CO_2_ in air with maximal humidity for subsequent development to blastocyst stage on day 7.

For vitrification, embryos were cryopreserved by using OPSs made in house as previously described.^14,16^ Briefly, a group of five embryos were first equilibrated in TCM199-HEPES+20% FBS, 10% ethylene glycol (EG) and 10% dimethyl sulfoxide (DMSO) for 3 min, and then transferred into the vitrification solution consisting of 20% FBS, 20% EG, and 20% DMSO, 0.5 M sucrose in TCM199-HEPES.^16^ Embryos were loaded into OPS and then directly plunged into LN_2_ within 1 min after exposure to the vitrification solution. Embryo warming was conducted by direct immersion of the OPS tip containing the embryos into 1 mL of 0.5 M sucrose in HEPES-buffered TCM199 supplemented with 20% FBS at room temperature. After 5 min in the 0.5 M sucrose solution, embryos were then rehydrated sequentially for 5 min in 0.25 M sucrose in HEPES-buffered TCM199+20% FBS and twice in TCM199-HEPES with 20% FBS. Warmed embryos were examined for immediate survival and cultured for subsequent development as described above.

### Experimental designs

The experiments described here are an extension of our previous efforts to cryopreserve porcine embryos in a closed system.^14^

**FIG. 1.**
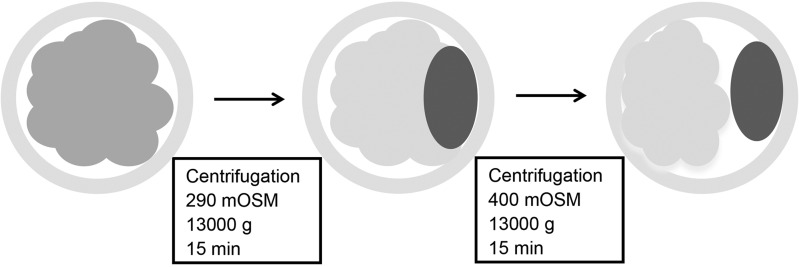
Schematic representation for the two-step centrifugation to externalize the intracellular lipids in porcine embryos.

Experiment 1 was designed to determine the efficiency of two-step centrifugation in combination with hypertonic treatment in Phase II centrifugation to delipidate embryos and the developmental potential of the delipidated embryos *in vitro*. Porcine zygotes were used in this experiment due to their relatively small perivitelline space. Porcine embryos are initially centrifuged for 15 min at 13,000 *g* to polarize intracellular lipids in 500 μL TL-HEPES supplemented with 7.5 μg/mL cytochalasin B and an osmolality of 290 mOSM. At the end of Phase I centrifugation, an equal volume of ∼530 mOSM TL-HEPES (made by adding fructose to 290 mOSM TL-HEPES) containing 7.5 μg/mL cytochalasin B was added to reach a final osmolality of ∼400 mOSM, and then the zygotes were immediately centrifuged for an additional 15 min. Polarized lipid droplets resulting from Phase I centrifugation were disassociated during the Phase II centrifugation due to the increased perivitelline space as a result of embryo shrinkage under hypertonic treatment (Fig. 1). After centrifugation, zygotes were assessed individually for the degree of lipid droplet separation under a dissecting microscope. Delipidated zygotes were then cultured *in vitro* to the blastocyst stage to assess their developmental competence compared to undelipidated zygotes.

Experiment 2 was designed to investigate the cryosurvival and subsequent developmental potential of day 2 porcine embryos in sealed straws after delipidation. Porcine embryos were delipidated and pre-equilibrated as described above, and then subjected to freezing in 0.25 mL straws. Control groups included OPS vitrified embryos after delipidation and noncryopreserved embryos without delipidation. Warmed embryos with an intact plasma membrane and zona pellucida were considered to be viable. Viable embryos were then cultured *in vitro* to the blastocyst stage to assess their developmental potential. Similar experiments were designed to investigate the cryosurvival and subsequent development of porcine day 3 and day 4 embryos (Experiment 3 and 4), respectively.

### Statistics

The percentage of embryo survival after cryopreservation and their subsequent development into blastocysts were analyzed by using a chi-squared test. A *p*-value less than 5% was considered to be significant.

## Results

**FIG. 2.**
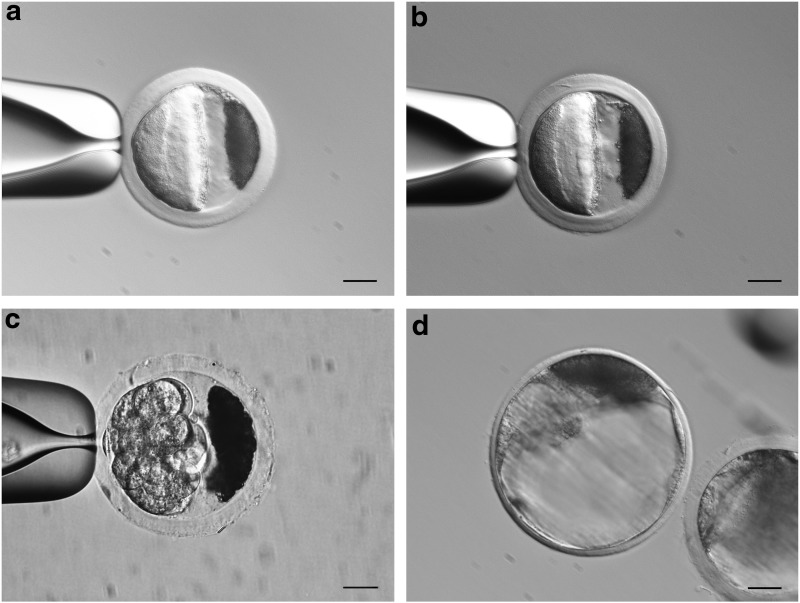
Representative images of porcine oocytes and embryos delipidated by the two-step centrifugation-based delipidation method: **(a)** mature oocyte; **(b)** day 1 embryo (zygote); **(c)** day 4 embryo (eight-cell stage); and **(d)** blastocyst resulting from delipidated day 1 embryo showing externalized lipid droplets remaining outside the blastomeres. Scale bars=20 μm.

By employing the two-step centrifugation-based delipidation method, intracellular lipid droplets in porcine zygotes were first polarized to one pole of the zona pellucida during the initial 15-min centrifugation in TL-HEPES with an osmolality of 290 mOSM, and then the polarized lipid droplets were externalized from zygotes after the second phase of centrifugation at 13,000 *g* for an additional 15 min in TL-HEPES at an osmolarity of 400 mOSM. This method is especially effective in delipidating oocytes and zygotes, which have a very small perivitelline space (Fig. 2a–d). The method resulted in a delipidation rate of 92.4% (73/79). Similar to other centrifugation-based delipidation methods, this modified method did not significantly compromise the developmental potential of delipidated embryos compared to undelipidated controls, assessed by their ability to develop into blastocysts *in vitro* (35.4% [29/79] vs. 35.1% [27/77], *p*>0.05).

On day 2, two- to four-cell-stage blastomeres of uniform size were selected for delipidation and cryopreservation. After cryopreservation by slow cooling and vitrification, the immediate survival of embryos was similar between the slow cooled and OPS vitrified groups (Table 1, chi-squared test, *p*>0.05). After culture *in vitro*, there was no significant difference in blastocyst rates between the two cryopreserved groups; however, they were significantly lower than the untreated control group (*p*<0.05).

**Table 1.** **Cryosurvival and Subsequent Development of Day 2 Porcine Embryos *In Vitro* After Two-Step Centrifugation-Based Delipidation and Cryopreservation**

Treatments					
Delipidation	Slow cooling	Vitrification	No. of embryos cryopreserved	No. of embryos survived	No. of blastocysts (%±SEM)
+	+	−	158	150 (94.9)^a^	17 (10.8±4.8)^a^
+	−	+	148	146 (98.6)^a^	8 (5.4±2.4)^a^
−	−	−	137	N/A	67 (48.9±1.9)^b^

Values with different superscript letters within columns indicate statistical differences (*p*<0.05, chi-square test).

N/A, not applicable.

Similarly, day 3 and 4 embryos were selected by using the same criterion as day 2 embryos and were then subjected to the two-step delipidation and cryopreservation. The results for immediate cryosurvival and subsequent development to blastocysts are shown in Tables 2 and 3, respectively. Within each experiment, there were no significant differences in the immediate survival rates between embryos cryopreserved by slow cooling and those by OPS vitrification (*p*>0.05). Embryos from the two cryopreserved groups were able to develop into blastocysts at similar rates (*p*>0.05). However, their developmental competence to blastocysts *in vitro* was significantly compromised compared to untreated controls (*p*<0.05).

**Table 2.** **Cryosurvival and Subsequent Development of Day 3 Porcine Embryos *In Vitro* After Two-Step Centrifugation-Based Delipidation and Cryopreservation**

Treatments					
Delipidation	Slow cooling	Vitrification	No. of embryos cryopreserved	No. of embryos survived	No. of blastocysts (%±SEM)
+	+	−	123	118 (95.9)^a^	23 (18.7±2.9)^a^
+	−	+	126	122 (96.8)^a^	18 (14.3±3.5)^a^
−	−	−	119	N/A	55 (46.2±5.9)^b^

Values with different superscript letters within columns differ significantly (*p*<0.05, chi-square test).

**Table 3.** **Cryosurvival and Subsequent Development of Day 4 Porcine Embryos *In Vitro* After Two-Step Centrifugation-Based Delipidation and Cryopreservation**

Treatments					
Delipidation	Slow cooling	Vitrification	No. of embryos cryopreserved	No. of embryos survived	No. of blastocysts (%±SEM)
+	+	−	85	80 (94.1)^a^	25 (29.4±7.1)^a^
+	−	+	85	79 (92.3)^a^	29 (34.1±3.8)^a^
−	−	−	95	N/A	45 (47.4±5.5)^b^

Values with different superscript letters within columns indicate statistical differences (*p*<0.05, chi-square test).

## Discussion

Efficient cryopreservation of early-stage porcine embryos with an intact zona pellucida in a closed system will enable efficient cryobanking and safeguard of swine model genetics. Our experiments are part of our continuing effort aiming at developing efficient protocols for cryobanking swine models. In this study, we employed a two-step centrifugation-based delipidation method to efficiently delipidate porcine embryos without damaging their zona pellucida. Porcine embryos at two-cell to morula stages were cryopreserved in a closed system by using the slow cooling method. The immediate survival and subsequent developmental rates to blastocysts were similar to those vitrified at ultrarapid cooling rates. However, the developmental potential of cryopreserved embryos was significantly reduced compared to the noncryopreserved controls. Since the overall efficiencies were low across the stages examined, further research is needed to improve the developmental potential of cryopreserved early-stage porcine embryos.

Porcine oocytes and preimplantation embryos have high levels of intracellular lipids that are demonstrated to be associated with their high sensitivity to low temperature.^18,20^ Delipidation through centrifugation before cryopreservation significantly improved the cryosurvival and subsequent development of porcine embryos at various stages of development and live piglets have also been obtained after the treatment.^18,20^ The original centrifugation-based mechanical delipidation method requires micromanipulation to remove the polarized lipid droplets to avoid their redistribution into the cytoplasm of the blastomeres.^9,18,26^ This procedure requires expensive equipment, trained personnel, and is also time-consuming. To simplify the procedure and avoid the damage to zona pellucida, several noninvasive delipidation procedures have been developed, including partial digesting of the zona pellucida before centrifugation, centrifugation under hypertonic conditions, or centrifugation at high speed for embryos at stages with large perivitelline spaces.^11,14,21,22^ Among these procedures, the delipidation rates ranged from 60% to 80%. With the modified two-step delipidation procedure in this experiment, the delipidation rate was significant higher than those achieved by previous procedures.

The centrifugation-based delipidation procedure effectively increases the cryosurvival and subsequent developmental competence for blastocyst-stage embryos. This treatment enables successful vitrification of porcine embryos at the blastocyst stage with intact zona pellucida in 0.25 mL ministraws and live piglets have resulted after surgical transfer of vitrified/warmed blastocysts.^14^ However, a similar approach has not been readily translated to the cryopreservation of early-stage porcine embryos. When day 2 porcine embryos were vitrified in 0.25 mL straws, the warmed embryos lost their potential to develop into blastocysts *in vitro* (data not shown). Instead, when slow cooling was employed, cryosurvival and blastocyst rates comparable to those vitrified at the ultrarapid cooling rate (OPS vitrification) have been achieved across embryos from day 2 to 4. The improved cryosurvival and subsequent development resulting from slow cooling may be partially due to the reduced osmotic stress encountered during slow cooling compared to vitrification. During vitrification, blastomeres undergo severe osmotic stress due to dramatic volume changes resulting from the high concentration of cryoprotectants. Osmotic stress has been demonstrated to cause irreversible damage to the cytoskeleton.^27^ However, the overall efficiencies for the cryopreservation of early-stage porcine embryos remained low across all the stages of embryos examined. Several other factors may also account for the low efficiencies.

Similar to other mammalian embryos, the porcine embryos' sensitivity to low temperature is also stage dependent. Early-stage embryos are more sensitive to low temperature than later stage embryos.^28,29^ The larger size of blastomeres in early-stage embryos make the cells more susceptible to cryoinjuries, such as osmotic stress.^30,31^ The few number of cells in early-stage embryos will also contribute to their cryosensitivity because early-stage embryos are less tolerant to the loss of a few blastomeres compared to later stage embryos.^31^ In addition, early-stage porcine embryos also have more and larger intracellular lipids compared to the blastocyst-stage embryos.^32,33^ Without prior delipidation, a significant percentage of early-stage porcine embryos generated *in vivo* lose their viability and subsequent developmental potential compared to those vitrified at the blastocyst stage; even with super fine OPS and Vit-Master vitrification used.^12^ Delipidation significantly improved the cryosurvival and developmental competence of cryopreserved embryos.^20,34,35^ Live piglets were obtained by transfer of frozen two- to four-cell-stage embryos derived *in vivo* after centrifugation-based mechanical delipidation and micromanipulation.^18^ Porcine embryos generated *in vitro* exhibit various abnormalities, including higher intracellular lipid contents, fewer cells, and fragmented blastomeres, and may also account for the reduced developmental competence.^36^ Moreover, utilizing more effective cryoprotectants may also improve the cryosurvival and subsequent development of *in vitro-*derived porcine embryos. For example, EG and propylene glycol have been demonstrated to be effective cryoprotectants for porcine oocytes.^37^ Therefore, optimization of the *in vitro* production system and cryopreservation protocol will contribute to the improvement of porcine embryo cryopreservation and the cryobanking of swine models.

In conclusion, complete disassociation of intracellular lipid droplets in early-stage porcine embryos has been achieved by using a two-step centrifugation-based method. Cryopreservation of day 2 to 4 embryos after delipidation by using slow cooling resulted in cryosurvival and subsequent development rates comparable to those cryopreserved by OPS vitrification. However, the overall development rates remain low and further research is needed to increase the efficiency of porcine embryo cryopreservation through improving the quality of embryos generated *in vitro* and optimization of cryopreservation protocols. The results reported here are part of our continuing efforts aiming at developing a protocol for the cryobanking of swine models. Since the efficiencies are low, our efforts will continue to focus on the optimization of protocols for embryo production and cryopreservation. The developmental competence of cryopreserved embryos *in vivo* will be assessed once the efficiency is significantly improved.

## Abbreviations Used

CRISPR: clustered regularly interspaced short palindromic repeats
DMSO: dimethyl sulfoxide
EG: ethylene glycol
FBS: fetal bovine serum
LN_2_: liquid nitrogen
mTBM: modified Tris-buffered medium
OPS: open pulled straw
PROH: 1,2-propanediol
